# Psychometric Properties of the Dental Activities Test: An Exploratory Factor Analysis in Older Adults with Cognitive Impairment

**DOI:** 10.1155/2018/8625916

**Published:** 2018-12-17

**Authors:** Xi Chen, Wen Liu

**Affiliations:** ^1^Department of Preventive and Community Dentistry, College of Dentistry, University of Iowa, Iowa City, Iowa, USA; ^2^College of Nursing, University of Iowa, Iowa City, Iowa, USA

## Abstract

**Objective:**

The Dental Activities Test (DAT) was developed to be used by dental, nursing, and other health professionals to assess the ability of persons with dementia to perform oral health-related activities and aid care planning. The instrument was designed as a unitary scale and has excellent internal consistency, test-retest reliability, interrater reliability, and construct validity. This study examines the underlying factor structure of the DAT among older adults in assisted living settings.

**Methods:**

In a secondary analysis of the data from the original study, the results of testing of 90 older adults with normal to severely impaired cognition from three assisted living communities in North Carolina from March 2013 to February 2014 were studied. An exploratory factor analysis was used to assess the dimensionality of the presumed unitary assessment scale.

**Results:**

Two-factor structures were explored. A one-factor model demonstrated acceptably mixed model fit, and a two-factor model had good model fit with moderate correlation between the two factors (*r* = 0.667, *p* < 0.05). All the items in the one-factor model demonstrated significant factor loadings (loadings ≥ 0.39, all *p* < 0.05), while the loadings of some items in the two-factor model (nonsignificant or cross-loadings, loadings < 0.40) did not meet the criteria of factor selection. The one-factor structure was preferred based on the criteria of Scree Plot, eigenvalue, and factor interpretability in relation to clinical relevance.

**Conclusions:**

The study provided preliminary evidence that the Dental Activities Test has a unidimensional construct among older adults with cognitive impairment. It suggested that this instrument can be used as a unitary scale to assess dental-related function in persons with dementia. Future testing, including using a confirmatory factor analysis, in a new sample is needed to further assess the usefulness and psychometric properties of this instrument.

## 1. Introduction

Functional impairment is a characterized symptom of dementia [1]. It is also an essential component of the diagnostic criteria of dementia [2]. Along with dementia progression, the ability to perform activities of daily living is interfered [3–6]. Dental-related function, which refers to the ability to perform oral health-related activities (e.g., brushing teeth, cleaning dentures, and use of fluoride or oral rinse as directed), can also be impaired and lead to poor oral hygiene [7, 8]. Poor oral hygiene, together with xerostomia, inadequate caregiver support, and the lack of regular dental care, increases the risk of dental caries and starts the cascade of oral health deterioration [9–12]. Oral disease and infection can in turn affect the quality of life, cause malnutrition, increase insulin resistance, and lead to recurrent respiratory infection, delirium, and other life-threatening conditions [13–17].

Given that impaired dental-related function plays an important role in the pathway from cognitive impairment to dental caries in persons with dementia [7, 8], it is essential to assess dental-related function impairment and incorporate its impacts on clinical treatment planning. To address this need, we developed and validated the Dental Activities Test (DAT), the first clinical tool specifically designed for dental, nursing, and other health professionals to measure dental-related function in persons with dementia [18]. The DAT consists of 9 oral health-related activities that are routinely performed by persons with dementia at home or at clinics. The test items include following instructions to determine a medication schedule, rinsing the mouth, opening and closing the mouth, moving the tongue as directed, brushing the teeth, putting toothpaste on a toothbrush, locating the lower front teeth with a finger, describing the status of the upper right front tooth, and making a decision about a hypothetical acute oral infection [18]. This assessment can be completed in 4-15 minutes (mean = 6, SD = 2) depending on the patient's cognitive status. It has excellent internal consistency (Cronbach alpha = 0.9), test-retest reliability (*r* = 0.84), and interrater reliability (*r* = 0.9) [18]. It is significantly associated with widely used cognitive and functional assessments, demonstrating good construct validity [18]. Using the Dental Activities Test with a total score ranging from 0 to 9, dental and other health professionals can, for the first time, reliably stage persons with dementia into 4 dental-related functional groups: independent (score 9), needs supervision (score 6-8), needs assistance (score 3-5), and full care (score 0-2). The objective nature of the assessment and categorization facilitates individualized, functionally tailored oral health interventions.

The reliability and construct validity of the Dental Activities Test have been demonstrated in our previous study using classical test theory [18], and yet some important psychometric properties have not been studied. For instance, although it was designed as a unitary scale, the underlying factor structure of the DAT remains unexamined. Consequently, whether dental-related function is better understood as a single, general factor (thus measured by a unitary scale) or as consisting of multiple, independent dimensions (measured by a scale with multiple subscales) remains unclear. Knowing the number and nature of factors that underlie the latent construct of dental-related function is important. It would help us understand whether we should use the whole battery or a subset of the DAT to assess dental-related function in persons with dementia. To address these questions, we preliminarily examined the construct validity and underlying structure of this presumed unitary scale using an exploratory factor analysis.

## 2. Materials and Methods

The present study was a secondary analysis based on an existing dataset that was collected during the psychometric testing of the DAT. The detailed study design, sampling method, and data collection protocol have been reported elsewhere and are briefly described below [18].

### 2.1. Study Participants

Residents were eligible for participation if they were 50 years of age or older, had no blindness and deafness or a severe physical disability (e.g., hemiplegia), and spoke English. Individuals with an oral health condition that required antibiotic prophylaxis prior to dental treatment and/or that required an immediate dental referral were excluded from the study. Ninety older adults who had normal to severely impaired cognition were recruited from three assisted living communities in North Carolina from March 2013 to February 2014. Study participants were mainly female (79.1%), with a mean age of 84 years. Sixty-eight percent of the participants were white, and 29.7% were black. The vast majority (73.6%) of the participants were previously or currently married. The mean length of stay in the facility was 2.8 years.

### 2.2. Data Collection

During the study, participants were asked to complete an oral exam, a dental-related functional assessment, a cognitive assessment, and an assessment of global function. After informed consent was obtained from the resident or his/her family member, an oral examination was first completed by a trained geriatric dentist. On average, the dentate participants had 19.25 teeth, of which 37.1% presented with cavitated caries. Twenty-five percent of the participants lost all their teeth. The mean debris index (DI) [19] and gingival index scores were 1.83 and 1.51, respectively, indicating poor oral hygiene among these individuals.

Within the following week, a trained research staff member, who was blinded to the oral exam results, visited the participants to assess their dental-related function using the 9-item Dental Activities Test (Table 1). During the assessment, persons with dementia were asked to complete each of the 9 test items as directed, and a trained rater observed and scored the performance of each activity using a binary (0 or 1) scale. One point is assigned for each activity if it is completed exactly as directed without help. The total score (ranging from 0-9) reflects a patient's overall dental-related function, with a higher score indicating higher function.

Following the assessment of dental-related function, cognitive status was assessed using cognition (measured by the Saint Louis University Mental Status (SLUMS) [20] and the Minimum Data Set Cognition Scale (MDS-COGS) [21]). After that, a trained research staff interviewed the caregiver of the participant to assess their daily function using the MDS activities of daily living (MDS-ADL) scale [22] and the Lawton Instrumental Activities of Daily Living (IADL) [23].

Most of the participants had some sort of impairment in performing oral health-related activities. The mean score of the Dental Activities Test was 5.86 (SD = 3.0). The SLUMS, which is more sensitive to mild cognitive impairment, generated a mean score of 8.2 and found 88.9% of the participants with dementia. The mean MDS-COGS score was 3.56, suggesting a mild-to-moderate cognitive impairment in the study participants. Functional assessments similarly revealed a range in abilities. The mean MDS-ADL and IADL scores were 11.4 (SD 9.6, range 0-32) and 4.8 (SD 3.0, range 0-12), respectively.

### 2.3. Statistical Analysis

Descriptive statistics were used to describe sample characteristics using SPSS 25.0 (SPSS, Chicago, IL). The frequency and percentage of each response option for the 9 test items were described. An exploratory factor analysis (EFA) using oblique rotation (geomin) was conducted to identify the initial factor structure of the DAT using Mplus 7.1 [24]. Oblique rotation allows the observed variables to be correlated and produces more realistic and statistically more appropriate factor structures than orthogonal methods [25, 26]. All the item data were treated as binary, and weighted least squares means and variance- (WLSMV-) adjusted estimation, which is appropriate for categorical data [24], was used as default. There were only 3 items with missing data (1.1%-5.6%) in the 9 test items, which were treated as missing data. Factor extraction was based on three criteria: eigenvalues greater than or equal to 1, the Scree Plot, and factor interpretability based on the content of the items [27]. To identify items loaded on each factor, the criterion of standardized factor loadings greater than 0.40 [28] was applied. Factor loadings less than 0.40 are considered weak, and factor loadings greater than 0.60 are strong [29].

Model fit indices were examined to determine how well alternative models fit the data. The indices included the chi-square goodness-of-fit index, root mean square error of approximation (RMSEA), comparative fit index (CFI), Tucker-Lewis index (TLI), and standard root mean square residual (SRMR). A nonsignificant chi-square test indicates a good fit. As chi-square is sensitive to large sample size, its significance should not be ignored but should be interpreted with caution [30]. CFI and TLI equal to or higher than 0.95 suggest an acceptable fit [30, 31]. An RMSEA of 0.08 or less and SRMR of 0.05 or less indicate reasonable errors of approximation, whereas an RMSEA between 0.08 and 0.10 indicates a mediocre fit [31, 32].

## 3. Results

### 3.1. Distribution of Item Responses

The distribution of responses of the 9 test items was described in Table 1. Participants' responses were skewed toward being independent in item 3 (open and close mouth) and item 7 (finding a tooth), less skewed in item 4 (tongue movement) and item 5 (tooth brushing), relatively evenly distributed in item 2 (rinse and spit), item 6 (putting toothpaste on a toothbrush), item 8 (perceiving an oral health condition), and item 9 (response to oral infection), and skewed toward being dependent in the screening item, item 1 (medication schedule). The distribution of item responses indicated that item 3 was most frequently endorsed among participants, followed by item 7, indicating that these items were relatively easy to perform among this population. Comparatively, the screening item (item 1) was least frequently endorsed by participants, indicating that this item was the most difficult to perform among this population.

### 3.2. Exploratory Factor Analysis

**Figure 1:**
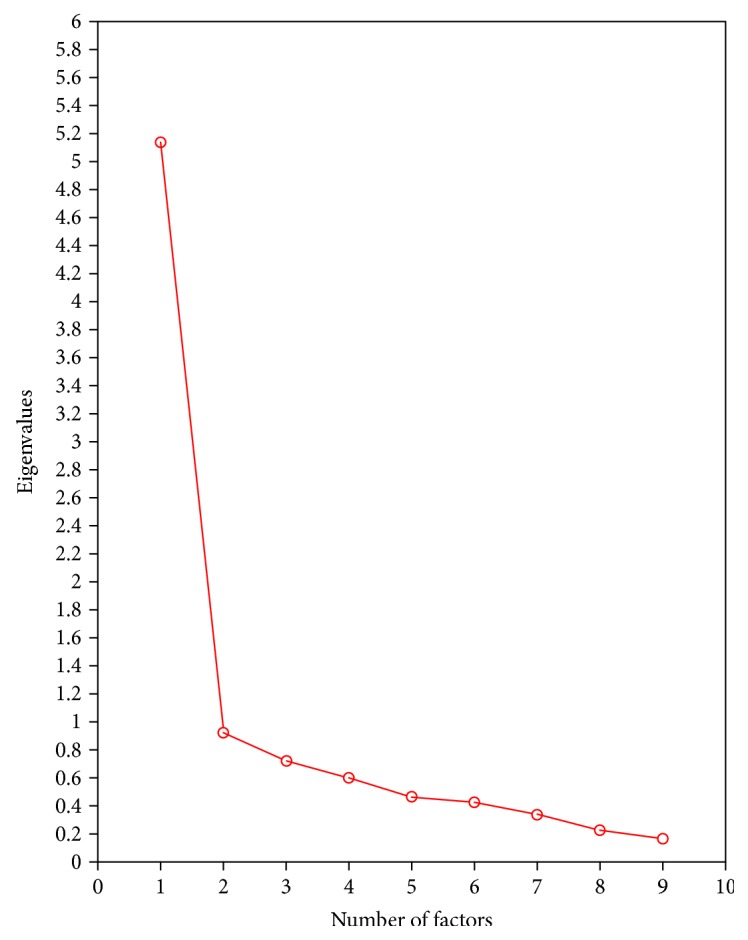
Scree Plot of the exploratory factor analysis on Dental Activities Test (*n* = 90).

One factor with eigenvalue greater than 1 (i.e., 5.136) was extracted, indicating a one-factor model based on Kaiser's criteria. The Scree Plot (Figure 1) also yielded one factor to be retained. Based on the fact that there are only 9 items in the DAT and the common rule that there should be a minimum of 3-item loading on one single factor, model fit indices for the one-factor model as well as for the two-factor model unrestricted between covariances were examined (Table 2). The one-factor structure showed a mixed model fit. A total of 55.65% of variance in dental-related function was explained by the one-factor structure. The two-factor structure demonstrated a good fit to the data with moderate correlation between the two factors (*r* = 0.667, *p* < 0.05).

Table 3 showed factor loadings for the one- and two-factor structures. All the items had significant loadings greater than 0.40 on the single-factor structure, except for the item “medication schedule” with a significant loading close to 0.40 (i.e., 0.39). In the two-factor model, six out of the nine items were loaded significantly on one of the two factors with a standardized loading greater than 0.40. Only one item, “finding a tooth,” was loaded significantly on the other factor. Two items, “tongue movement” (i.e., 0.45 and 0.38) and “response to oral infection” (i.e., 0.32 and 0.34), were loaded nonsignificantly on both factors. Even though this two-factor structure had a better model fit, it did not seem valid based on the findings that there were only two items in one factor and one item with a loading lower than 0.40 and nonsignificant loadings on both factors. The one-factor structure was preferred based on the content of the items, clinical relevance, and standardized factor loadings.

## 4. Discussion

When using the Dental Activities Test in clinical practice, whether we can use a subset of this instrument to assess a particular dental-related functional domain (e.g., oral self-care function) was frequently raised. In response to this question, we conducted this analysis to examine the underlying factor structure of this presumed unitary scale among older adults living in assisted living settings using the EFA modeling approach. It revealed a single-factor structure for this scale, suggesting the unidimensionality of the construct of dental-related function. The findings of this study provided additional evidence for the DAT scale as a structurally valid measure of dental-related function among persons with dementia and suggested that a functional assessment based on one or a few items of the Dental Activities Test is inappropriate and not recommended.

The results of this study showed that a single-factor structure is preferable to a two-factor structure. In the single-factor EFA model, all the 9 items clustered together well, indicating that there were strong interrelations among the items and that the total score of the 9 items represents the level of dental-related function. All the items had positively strong factor loadings except for item 1 “determining a medication schedule following instructions” with a loading close to 0.40. As shown in Table 1, this item is the most difficult item. It has a strong cognitive potency and requires high level of cognitive function to accomplish, which differentiates this item from other test items. Our previous study showed that the removal of this item increased Cronbach's alpha only by 0.008 [18]. Because this item seems to address a component of DRF not clearly evident in the other items and significantly contributes to the single-factor structure, it was retained in the model. This item provides a full conceptualization of dental-related function and is relevant for the vast majority of persons with dementia living in community [33] where self-management of fluoride toothpaste, oral rinse, or other dental-related medications is more prevalent.

Keeping item 1 in the model can also help address one potential limitation of the Dental Activities Test. A survey of special care dentists revealed that a dental-related functional assessment should be less than 5 minutes to be useful in a dental practice [34]. On average, this assessment requires 6 minutes to complete, slightly longer than the preferred time frame of dental professionals. And yet it only takes 30-40 seconds for cognitively intact persons or 1-2 minutes for those with impairment to complete item 1, the screening item [18]. Failure on this item suggests that the patient is highly likely to have impaired dental-related function [18] and requires a complete assessment to stage his/her function and develop a stage-appropriate treatment plan. Therefore, using item 1 as a screening item can enable clinicians to quickly identify functionally dependent patients and reallocate time and effort to these individuals, addressing dental professionals' concern on the administration time of the DAT.

This study provides preliminary information to support a unidimensional construct of the Dental Activities Test, which is clinically meaningful. First, it confirms that a summative score can be used to reflect the global function of persons with dementia in performing oral health-related activities. Based on the score, these individuals can be categorized into one of the four groups for dental-related function: independent, need supervision, need assistance, and full care [18]. With this information, functionally tailored oral care plan can then be developed to achieve the desired clinical outcomes [7]. Moreover, this finding also suggests that when assessing dental-related function in persons with dementia, the whole battery of the Dental Activities Test should be used. Previous studies show that dental professionals tend to assess the ability of persons with dementia to perform oral health-related activities by asking these patients to demonstrate oral care instructions [34]. Although it is quick, this unstructured approach can neither provide a completed functional profile for the examinee nor accurately reflect his/her ability to follow homecare instructions and perform oral self-care activities. It is therefore hard to plan for individualized care based on this incomplete information and is not recommended to use this approach for functional assessment.

This study revealed the underlying factor structure of the Dental Activities Test, and yet it was limited by virtue of being a secondary data analysis. The sample was mostly women, and thus future testing of the single-factor structure is needed with a sample with more balanced gender distribution. The sample size was relatively small. While it is sufficient for an exploratory factor analysis (e.g., the sample-to-item ratio was 10 to 1) [35, 36], a confirmatory factor analysis was not performed due to small sample size. Future work is needed to validate the one-factor structure among older population to confirm the unidimensionality of this instrument. Also, the sample did not include residents from other settings such as community and skilled nursing or rehabilitation settings, and future work may benefit to validate the single-factor structure among persons with dementia residing in different settings.

In conclusion, the study provided preliminary evidence that the Dental Activities Test has a unidimensional construct among older adults with cognitive impairment. Future testing using a confirmatory factor analysis is needed to confirm and validate this factor structure among this population.

## Figures and Tables

**Table 1:** Percent of participants responding correctly to individual DAT items.

	Percent of participants responding correctly to the item (*N* = 90)
Item 1: determining a medication schedule following instructions	19
Item 2: rinsing mouth	66
Item 3: opening and closing mouth	87
Item 4: moving tongue	73
Item 5: brushing teeth—person with teeth or denture	72
Item 6: putting toothpaste on a toothbrush	62
Item 7: locating the lower front teeth with a finger	78
Item 8: describing the status of the upper right front tooth	63
Item 9: making a decision about a hypothetical acute oral infection	64

**Table 2:** Model fit statistics for one-factor and two-factor models (*n* = 90).

Fit indices (model fit criteria)	One-factor model	Two-factor model
*x* ^2^ (df), *p* (nonsignificant)	58.31 (27), *p* < 0.001	20.17 (19), *p* = 0.38
RMSEA (90% CI), *p* (<0.08)	0.11 (0.07, 0.15), *p* < 0.01	0.03 (0.00, 0.09), *p* = 0.63
CFI (>0.95)	0.93	0.99
TLI (>0.95)	0.90	0.99
SRMR (<0.05)	0.05	0.03

Note: df = degrees of freedom; RMSEA = root mean square error of approximation; CI = confidence interval; CFI = comparative fit index; TLI = Tucker-Lewis index; SRMR = standard root mean square residual.

**Table 3:** Standardized factor loadings for EFA models.

Items	One-factor model	Two-factor model	
	1	1	2
Item 1: medication schedule	0.393^∗^	−0.070	**0.467** ^∗^
Item 2: rinse and spit	**0.692** ^∗^	−0.023	**0.726** ^∗^
Item 3: opening and closing mouth	**0.696** ^∗^	0.314	**0.426** ^∗^
Item 4: tongue movement	**0.765** ^∗^	**0.454**	0.385
Item 5: tooth brushing	**0.819** ^∗^	0.028	**0.822** ^∗^
Item 6: putting toothpaste on a toothbrush	**0.848** ^∗^	0.080	**0.820** ^∗^
Item 7: finding a tooth	**0.820** ^∗^	**1.077** ^∗^	0.000
Item 8: perceiving an oral health condition	**0.762** ^∗^	−0.042	**0.821** ^∗^
Item 9: response to oral infection	**0.605** ^∗^	0.324	0.338

Note: all the factor loadings larger than 0.40 are bold. ^∗^*p* < 0.05.

## Data Availability

The data used to support the findings of this study are available from the corresponding author upon request.
